# Management of Vasoproliferative Tumors of the Retina with Macular Complications by Pars Plana Vitrectomy Combined with Episcleral Cryotherapy

**DOI:** 10.1155/2021/6667755

**Published:** 2021-04-13

**Authors:** Wenhua Zhang, Zeyuan Qiang, Hao Song, Xiaoli Li, Handong Dan, Keke Ge, Pan Li, Zixu Huang, Dongdong Wang, Feng Chen, Bin Zheng, Zongming Song

**Affiliations:** ^1^Henan University People's Hospital, Henan Provincial People's Hospital, Henan Eye Hospital, Zhengzhou 450003, Henan, China; ^2^Zhengzhou University People's Hospital, Henan Provincial People's Hospital, Henan Eye Hospital, Zhengzhou 450003, Henan, China; ^3^Henan Eye Institute, Henan Eye Hospital, Henan Provincial People's Hospital and Henan University People's Hospital, Zhengzhou 450003, Henan, China; ^4^Eye Hospital, Wenzhou Medical University, Wenzhou 325000, Zhejiang, China

## Abstract

**Objective:**

To evaluate the efficacy of pars plana vitrectomy (PPV) combined with episcleral cryotherapy in treating vasoproliferative tumors of the retina (VPTR) with macular complications.

**Methods:**

In this retrospective noncomparative interventional case-series analysis, we included 11 eyes of ten patients diagnosed with VPTR. All patients underwent comprehensive ophthalmic examinations and were treated with PPV combined with episcleral cryotherapy. Best-corrected visual acuity (BCVA), tumor activity, retinal morphological structure, and postoperative complications were evaluated.

**Results:**

Macular complications included epimacular membrane (*n* = 10), macular hole (*n* = 3), and macular edema (*n* = 1). Tumors were treated with triple freeze-thaw episcleral cryotherapy during PPV. The mean logarithm of minimal angle of resolution (logMAR) BCVA dropped from 0.62 ± 0.58 to 0.39 ± 0.46. The difference between the mean values of logMAR BCVA before and after treatment was statistically significant (*t* = 2.48, *P*=0.033). The tumor activity was controlled effectively in nine cases. Compared with preoperative tumor activity, tumor activity after treatment was significantly lower (*P* < 0.01). The increase of central retinal thickness and the disruption of retinal layers were associated with macular holes, macular edema, and retinal proliferative membrane. After the treatment, visual acuity improved in 91% of the cases, and 73% had no long-term complications.

**Conclusion:**

PPV combined with episcleral cryotherapy promoted tumor regression, preserved retinal integrity, and improved visual acuity. Thus, the combination of PPV with episcleral cryotherapy can be considered effective and safe for the management of VPTR with macular complications.

## 1. Introduction

Vasoproliferative tumors of the retina (VPTR) were first described by Henkind et al. [1] in 1966 as a rare benign disease often causing severe visual impairment. By analyzing the clinical characteristics of 103 VPTR patients, Shields et al. [2] proposed VPTR classification guidelines based on the clinical presentation (idiopathic or secondary). Patients with early-stage VPTR have inconspicuous symptoms, and macular invasion at later stages of the disease may cause hemorrhage or exudation. VPTR complications, such as epimacular membranes, macular holes, vitreous hemorrhage, and exudative retinal detachment, are predominant causes of vision impairments [3]. VPTR etiology and histopathological characteristics are essential factors for determining optimal treatment. However, VPTR pathogenesis and histopathological features remain unclear.

Although retinal neovascularization has been proposed as a critical feature of VPTR, Poole et al. [4] reported that conservative treatment to inhibit angiogenesis enhanced glial cell proliferation. Shields et al. [5] suggested that VPTR are caused by reactive vascular hyperplasia due to retinal ischemia or other retinal insults. Recently, VPTR have been reported in patients with neurofibromatosis type 1 [6]. Zheng et al. found that VPTR were confined to the inner retina and had profound vascularization. They also reported that VPTR were often accompanied by cellulose exudation and inflammatory cell infiltration [7]. Currently, VPTR are considered to be benign, slow-growing lesions.

Various VPTR treatment methods have been reported. These methods include cryotherapy, plaque radiotherapy, surgical tumor resection, laser photocoagulation, photodynamic therapy, vascular endothelial growth factor (VEGF) inhibition, and surgical vitrectomy [8, 9]. In clinical studies, conservative VPTR treatment provided limited clinical benefits to patients, and most of whom had to undergo surgery due to complications. In this study, we retrospectively analyzed the clinical characteristics of patients before and after treatment for VPTR and found that pars plana vitrectomy (PPV) combined with episcleral cryotherapy was safe and effective in patients with macular complications due to VPTR.

## 2. Materials and Methods

All patients diagnosed with VPTR and treated with PPV combined with episcleral cryotherapy at the Henan Provincial People's Hospital and the Eye Hospital of Wenzhou Medical University from January 2011 to August 2020 were included in the retrospective review. Ten patients (11 eyes) met the inclusion criteria. Visual impairment and VPTR complications were present in all patients. This study adhered to the principles of the Declaration of Helsinki. Approval was provided by the Medical Ethics Committee of Henan Eye Hospital. All patients signed informed consent.

### 2.1. Related Inspection and Devices

Each eye had a comprehensive ophthalmic examination, including the best-corrected visual acuity (BCVA), medical optometry, slit-lamp biomicroscope, intraocular pressure (IOP), fundus photographs, fundus fluorescein angiography (FFA), A- and B-scan ultrasonography, axial length (AL), and spectral-domain optical coherence tomography (SD-OCT).

### 2.2. Inclusion and Exclusion Criteria

**Figure 1:**
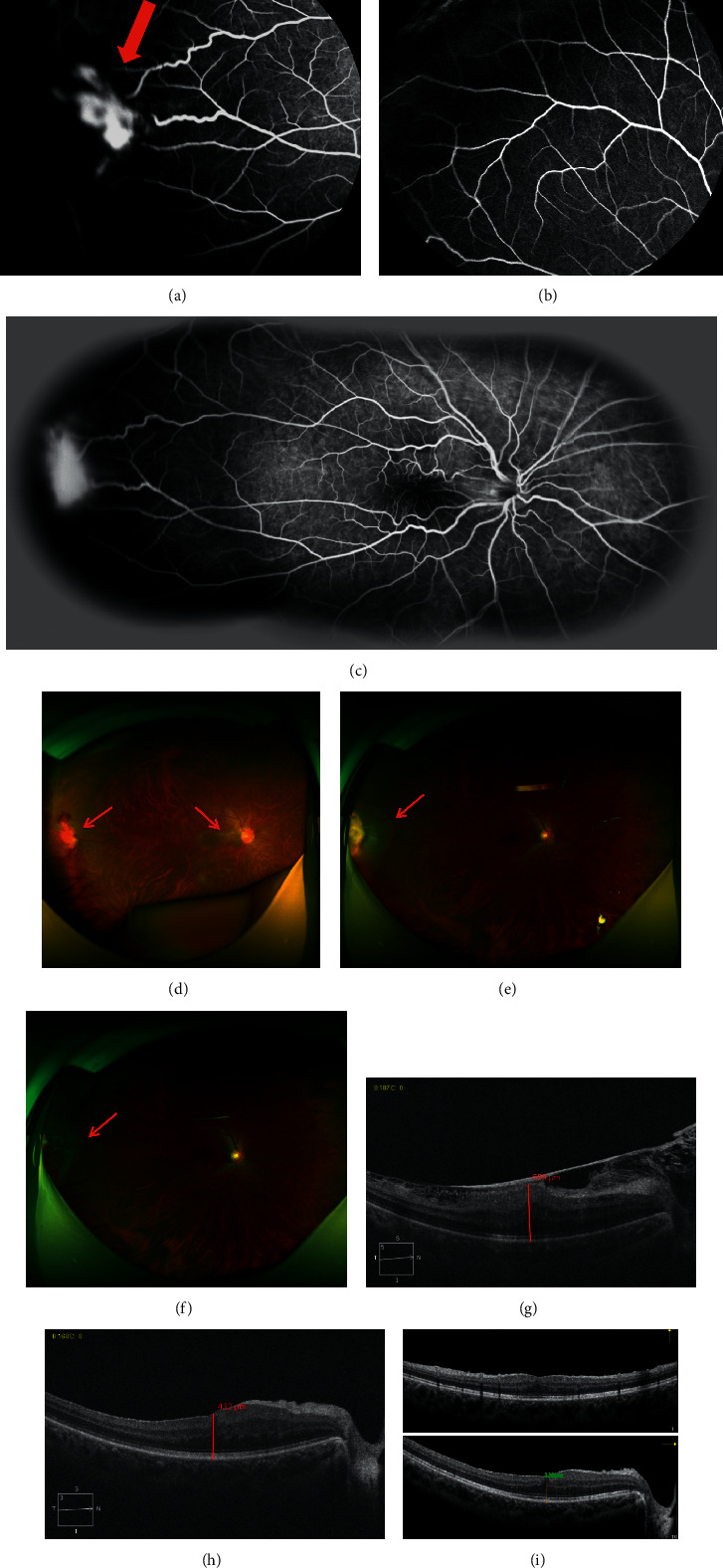
Right eye of a 30-year-old woman with vasoproliferative tumor of the retina and an epiretinal membrane (Case 9). Fundus fluorescein angiography (FFA): (a) (35.93 seconds) two tortuous and dilated blood vessels (feeder and draining vessels) were observed around the tumor. The early filling of the tumor was conspicuous. (b) (46.59 seconds) No perfusion areas around the fundus were observed. (c) (4 minutes 56.43 seconds) The apparent fluorescence leakage could be observed in the retina. Fundus photograph: (d) before treatment, the epimacular membrane was located in the temporal side of optic disc and an orange-red hemorrhagic mass sized 1.5 optic disc diameter (PD) could be observed in the temporal periphery. The best-corrected visual acuity (BCVA) was 0.7 logMAR. (e) One week after treatment with cryotherapy, laser spots were visible around the temporal retina. The peripheral retinal mass was fibrotic and yellowish. Feeder vessels were narrowed. (f) One month after treatment with cryotherapy, the tumor was regressive and surrounded by laser spots. BCVA was 0 logMAR. Spectral-domain optical coherence tomography (SD-OCT): (g) before treatment, the retinoschisis was caused by the epimacular membrane. The central retinal thickness (CRT) was 555 *μ*m. (h) One week after treatment, the retinal morphology was abnormal with macular edema. The CRT was 432 *μ*m. (i) One month after treatment, the retinal morphology was significantly improved. Mild edema and thickened retinal nerve fiber layers were shown in the SD-OCT. The CRT was 338 *μ*m.

**Figure 2:**
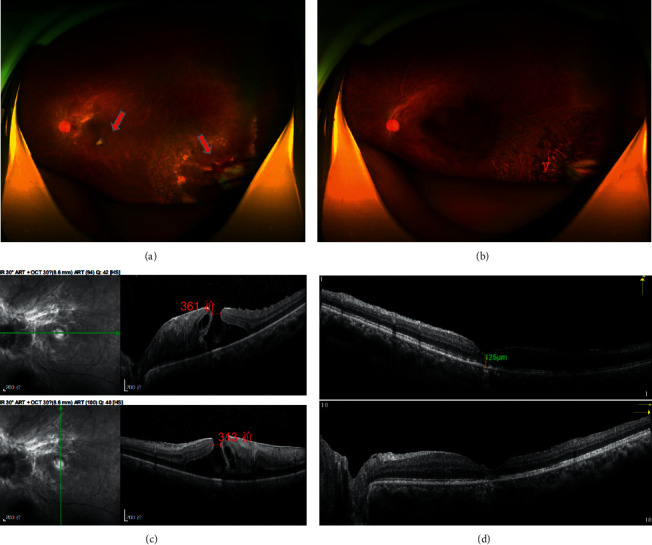
Left eye of a 31-year-old man with vasoproliferative tumor of the retina (Case 10): Fundus photograph: (a) before treatments, the macular hole with the size of 1/2 optic disc diameter (PD) could be seen in the macula. A yellow globular pale mass sized 5 PD was located in the inferior temporal periphery. The mass was connected with feeder vessels and was surrounded by laser spots. (b) Three months after cryotherapy, the tumor was depauperate with the occluded feeder vessels. Spectral-domain optical coherence tomography (SD-OCT): (c) before treatments, the epimacular membrane and an IV macular hole with a size of 361 *μ*m^*∗*^313 *μ*m could be seen. The best-corrected visual acuity (BCVA) was 1 logMAR. (d) Three months after treatments, the morphology of the macula was improved. The central retinal thickness was 125 *μ*m. The BCVA was 0.8 logMAR.

Globular pale masses with clear boundaries were diagnosed as VPTR. Slightly enlarged, tortuous vessels were frequently seen around the lesion, often accompanied by retinal exudation and vitreous hemorrhage (Figures 1 and 2). On FFA, strong fluorescence signals were observed in the retinal vascular mass and its feeder vessels. Fluorescein leakage was visible in the late frames (Figure 1). A- and B-scan ultrasonography indicated local thickening of the sphere wall of the tumor region. SD-OCT was used to assess retinal morphology and macular lesions (Figures 1 and 2). Patients with VPTR-related macular complications, such as epiretinal membrane, macular hole, and macular edema, were included in our analyses. Additionally, included patients had surgical indications and underwent PPV combined with episcleral cryotherapy. The postoperative follow-up time was more than three months. Patients who had not been treated with PPV combined with episcleral cryotherapy or without macular complications were excluded. Patients who were not followed-up or with incomplete data were also excluded.

### 2.3. Tumor Activity Was Determined by the Following Criteria

(1) Fluorescent leakage at the tumor site by the FFA; (2) subretinal effusion or exudation around the tumor, which did not subside during the follow-up period; (3) hemorrhage or membranous proliferation around the tumor, causing or aggravating ocular complications; and (4) presence of blood vessels around the tumor or the lesion. After treatment, slit-lamp and fundus examination, as well as fundus photography, was used to evaluate the area around the lesion. In the presence of signs of tumor activity, such as retinal neovascularization, retinal hemorrhage, retinal detachment, retinal exudation, or epiretinal membranes, FFA was performed.

### 2.4. Visual Acuity Calculation Method

BCVA was calculated as the logarithm of minimal angle of resolution (LogMAR), with light perception corresponding to 2.6 LogMAR, hand motions corresponding to 2.3 LogMAR, and counting fingers reaching 1.85 LogMAR [10].

### 2.5. Operation Method

Retrobulbar anesthesia was performed by a mixture of 2% lidocaine and 0.75% bupivacaine. As all patients had macular complications and required intraocular surgery, 23- or 25-gauge PPV was performed. In all patients, tumors were treated with triple freeze-thaw episcleral cryotherapy during intraocular surgery. VPTR-associated ERM and other proliferative tissues were peeled. In patients with macular holes, the internal limiting membrane ILM was peeled. Laser photocoagulation was applied on the feeder vessels to close them, thereby promoting ischemia and contributing to tumor atrophy. The tamponade was injected intraocularly, and a mixture of dexamethasone and amikacin was injected into the subconjunctival sac to prevent the inflammation.

### 2.6. Statistical Analysis

SPSS 21.0 software was used for all statistical analyses. Levene's test was used to test for homogeneity of variance, and the Shapiro Wilk test was used to assess data normality. Data were expressed as mean values and standard deviations. Differences between two groups were analyzed using the paired *t*-test or Fisher's exact probability test. *P* values less than 0.05 were considered statistically significant.

## 3. Results

In this study, 11 eyes (left, *n* = 4; right, *n* = 7) from ten patients (one man and nine women) were retrospectively analyzed. The mean age of patients at the time of diagnosis was 49.00 ± 12.28 years. Of the ten patients, one was binocular, and the rest were monocular. Of the 11 eyes, ten were idiopathic VPTR, and the other one was secondary uveitis. Visual acuity was impaired in ten eyes (91%), muscae volitantes occurred in four eyes (36%), and retinal exudation was observed in three eyes (27%). Visual deformities (*n* = 1), visual field defects (*n* = 1), flash sensation (*n* = 1), and red eye (*n* = 1) were also observed. Before treatments, the average intraocular pressure (IOP) was 16.19 ± 1.12 mmHg, and the AL was 23.17 ± 0.22 mm. BCVA ranged from 0 to 2, with an average of 0.62 ± 0.58 LogMAR. The duration of symptoms ranged from one week to three years, with an average of 10.21 ± 4.01 months. Tumors were located in the inferior temporal region (64%), the superior temporal region (18%), the inferior nasal region (9%), and the inferior midperipheral retina (9%). The size of tumors ranged from 1.5 optic disc diameter (PD) to 6 PD, with an average of 3.32 ± 1.74 PD. The characteristics of patients and tumors are shown in Table 1.

The following complications were recorded: epimacular membrane (91%), macular hole (27%), and macular edema (9%). Tumor feeder vessels were tortuous and dilated. They could be observed in all of the cases. Laser photocoagulation was effective in closing the feeder vessels. Fundus photographs showed that after treatment, feeder vessels were occluded and regressive. Operation methods for all cases are summarized in Table 2. The average follow-up time was 15.64 ± 12.29 months (range, 3–38 months). LogMAR BCVA and the changes of logMAR BCVA at the last follow-up were 0.39 ± 0.46 and −0.23 ± 0.93, respectively. The statistical values of logMAR BCVA before and after treatments were significantly different (*t* = 2.48, *P*=0.033). Before the treatment, all tumors were active. After the treatment, tumors were active in two eyes (18%). The rest nine eyes (82%) were inactive. The number of active tumors before and after treatments was significantly different (*P* < 0.01) (Table 3). During the follow-up period, two patients developed epiretinal membrane, and one had retinal exudation; hence, the incidence of postoperative complications was 27%. Since all patients had VPTR complications before surgery, severe abnormalities in retinal morphology were observed. PPV combined with episcleral cryotherapy restored the macular morphology and foveal thickness in all patients.

## 4. Discussion

VPTR are rare and often misdiagnosed or missed. Thus, patients with VPTR often develop complications. In this study, we reviewed the characteristics of 11 eyes with VPTR from patients with macular complications. We found that PPV combined with episcleral cryotherapy not only improved vision but also promoted tumor regression.

Abolfathzadeh et al. [11] and Para-Prieto et al. [12] reported that extrascleral brachytherapy benefited patients with large tumors or extensive subretinal fluid. In such cases, extrascleral brachytherapy can be considered as a first-line treatment [13]. Chen reported a case of VPTR with epimacular membrane cured by intravitreal injection of bevacizumab alone [14]. Rogers et al. showed that intravitreal injection of bevacizumab temporarily reduced the thickness of some tumors [15]. However, the changes in tumor thickness and visual acuity were not statistically significant, and intravitreal bevacizumab injection alone failed to cause long-term regression of lesions. Moreover, intravitreal injection of bevacizumab may benefit patients with VPTR-related retinal neovascularization or exudative retinal changes caused by VEGF secreted by VPTR [16]. Laser photocoagulation can mitigate retinal exudation in patients with VPTR and retinal telangiectasia. In most cases, slit-lamp laser photocoagulation is sufficient, although direct endoscopic photocoagulation may be required in severe cases [17].

Manjandavida et al. demonstrated that transconjunctival double freeze-thaw cryotherapy (under local anesthesia using visualization with indirect ophthalmoscopy) was effective in patients with VPTR with a tumor size of 6 mm or less. After cryotherapy, VPTR-associated epiretinal membrane spontaneously regressed in 63% of cases without surgical intervention [18]. The findings of this study suggest that double freeze-thaw cryotherapy can prevent tumor progression to some extent; nevertheless, 37% of patients require secondary surgery to treat residual tumors. Garcia-Arumi et al. assessed 31 eyes with VPTR and vision-threatening complications. Triple freeze-thaw cryotherapy was performed using indirect ophthalmoscopy if laser photocoagulation was insufficient to render the tumor ischemic. Most patients with VPTR required surgery as initial treatment [19]. In this study, tumors were treated with triple freeze-thaw episcleral cryotherapy. Compared with retinal capillary hemangioma, feeder vessels of VPTRs were inconspicuous. In some patients, multiple feeder vessels were observed. Blocking the blood supply of tumors prevented the progression of VPTR. From our results, tumors in nine eyes (82%) were shrunk and presented activity loss. All VPTR-associated complications can be controlled. These results may indicate that the triple freeze-thaw episcleral cryotherapy is more beneficial to completely inactivate the tumor.

VPTRs were recognized as benign, slow-growing lesions. In order to maintain retinal morphology and reduce the occurrence of postoperative complications, we did not choose to resect the tumor. Triple freeze-thaw episcleral cryotherapy led to tumor shrinkage and reduction in vascularization. Additionally, vitrectomy alleviated macular membrane and vitreous hemorrhage. Patients with macular holes were also injected with a viscoelastic solution to protect the inner pigment epithelium (RPE). Visual acuity improved significantly in 10 of 11 eyes. No long-term complications were observed in eight eyes; however, two eyes had retinal proliferative membrane seven months after the operation, and one patient had slightly retinal exudation. Compared with simple tumor cryotherapy or tumor resection, this combined therapy may be more safe and effective.

Our study had certain limitations. First, due to the scarcity of this tumor type and the variety in treatment methods, the cohort size was limited. Second, we did not address the tumor volume before and after treatment because our goal was to treat the visual impairment and complications caused by the tumor rather than targeting the tumor itself. Finally, we did not compare PPV combined with episcleral cryotherapy with other techniques because our primary goal was to demonstrate the feasibility of this method.

In conclusion, VPTR should be treated according to the clinical presentation of patients. In patients with macular disease, PPV combined with episcleral cryotherapy can significantly improve visual acuity, eliminate tumor activity, reduce tumor recurrence, maintain retinal integrity, and accelerate postoperative recovery. Therefore, PPV combined with episcleral cryotherapy is an effective and safe method to treat VPTR.

## Figures and Tables

**Table 1:** Summary of patients' clinical characteristics.

Case no.	Sex	Age (y)	Eye	Symptom	Cla.	AL (mm)	IOP (mmHg)	TS (PD)	TL	Follow-up (month)	BCVA (LogMAR)		Tumor activity		Macular complication	
											Preoperation	Postoperation	Preoperation	Postoperation	Preoperation	Postoperation
1	F	55	R	DV	P	21.61	16.6	2	IT	19	0.7	0.2	Yes	No	EMM	
2	F	42	R	Fl; met	P	22.5	14	4	IT	8	0.3	0.2	Yes	No	EMM	
3	F	42	L	DV	P	23.92	16.4	2	SN	38	0.1	0	Yes	No	EMM; TRD	
4	F	58	R	DV; Re	S	23.14	10.3	2	IT	31	2	1.3	Yes	Yes	EMM; Uv; RE; SRF	
5	F	64	R	DV; Fl; FOL	P	22.84	16.3	6	ST	6	0.7	1	Yes	Yes	TRD; EMM	RE
6	F	64	L	DV	P	22.5	20.8	2	IT	6	0.15	0.1	Yes	No	EMM	EMM
7	F	60	L	DV	P	23.87	12.3	4	IT	17	0.2	0.1	Yes	No	MH; EMM; TRD	EMM
8	F	43	R	Fl	P	23.57	11.7	2	ST	6	0	0	Yes	No	VH; ME	
9	F	30	R	DV; RE	P	23.7	19.6	1.5	IT	30	0.7	0	Yes	No	EMM; REx	
10	M	31	L	DV; VFD; RE	P	23.66	20.3	5	IT	8	1	0.8	Yes	No	EMM; MH	
11	F	50	R	DV; RE	P	23.56	19.8	6	IM	3	1	0.6	Yes	No	EMM; MH	

F : female; M : male; R : right; L : left; DV : decreased vision; Fl : floater; Met.: metamorphopsia; Re : red eye; FOL : flash of light; RE : retinal exudation; VFD : visual field defect; Cla.: classification; AL : axial length; IOP : intraocular pressure; TS : tumor size; PD : optic disc diameter; TL : tumor location; IT : inferotemporal; SN : subnasal; ST : supratemporal; IM : inferior midperiphery; BCVA : best-corrected visual acuity; LogMAR : logarithm of minimal angle of resolution; EMM : epimacular membrane; TRD : tractional retinal detachment; Uv : uveitis; SRF : subretinal fluid; MH : macular hole; VH : vitreous hemorrhage; ME : macular edema.

**Table 2:** Summary of patients' surgical procedures.

Case no.	Sex	Age (y)	Eye	PRT	Phacoemulsification	ILMP	Electric coagulation	Tamponade
1	F	55	R	Laser-photo.	+	+	−	C2F6
2	F	42	R	EP-cryo.	−	+	−	BBS + TA
3	F	42	L	EP-cryo.	−	+	+	C3F8
4	F	58	R	EP-cryo.	+	+	−	BBS + TA
5	F	64	R	Laser-photo.	+	−	+	BBS + TA
6	F	64	L	EP-cryo.	+	+	−	BBS + TA
7	F	60	L	Laser-photo.	+	+	−	C3F8 + TA
8	F	43	R	Laser-photo.	−	-	−	SA
9	F	30	R	Laser-photo.	−	+	+	SA + TA
10	M	31	L	Laser-photo.	−	+	−	SO
11	F	50	R	Laser-photo.	−	+	−	C3F8

F : female; M : male; R : right; L : left; PRT : peritumoral retina treatment; Laser-photo.: laser photocoagulation; EP-cryo.: episcleral cryotherapy; ILMP : internal limiting membrane peeling; SA : sterilized air; TA : triamcinolone acetonide; SO : silicone oil.

**Table 3:** Comparison of BCVA and tumor activity of preoperation and postoperation.

	BCVA (logMAR)	Tumor activity (Yes/No)
Preoperation	0.62 ± 0.58	11/0
Postoperation	0.39 ± 0.46	2/9
	*t* = 2.48	Fisher
*P*	0.033	＜0.01

BCVA : best-corrected visual acuity; LogMAR : logarithm of minimal angle of resolution.

## Data Availability

The data of all patients were collected from clinical patients from Henan Provincial People's Hospital and Eye Hospital of Wenzhou Medical University from January 2011 to August 2020.
